# Navigating the Antiretroviral Therapy Switch Conundrum: Unveiling the Dilemma of Drug Resistance and Disease Progression in HIV/AIDS

**DOI:** 10.7759/cureus.56441

**Published:** 2024-03-19

**Authors:** Ankita Sharma, Gyan Vardhan, Puneet Dhamija, Vikas Kumar

**Affiliations:** 1 Prosthodontics, Adesh Institute of Dental Sciences and Research, Bathinda, IND; 2 Pharmacology, All India Institute of Medical Sciences, Rishikesh, Rishikesh, IND; 3 Pharmacology, All India Institute of Medical Sciences, Bathinda, Bathinda, IND

**Keywords:** arv resistance, art switch, virologic suppression, hiv aids, antiretroviral (arv)

## Abstract

There is a need to establish consensus for harmonization in antiretroviral (ARV) therapy (ART) switch treatment strategy and address the dilemma that exists in terms of subpar immune response to therapy or an immunologic deterioration while on therapy. The purpose of this review is to identify the factors that contribute to ARV treatment failure, such as insufficient dosage, drug interactions, poor adherence, drug resistance, and poor medication absorption. It is crucial to adopt a more efficient strategy to address this challenging dilemma. After ARV treatment failure, the aim of therapy is virologic suppression, which targets plasma viral load below the limits of detection as assessed by very sensitive tests with lower limits of quantification of 20 to 75 RNA copies/ml. The therapeutic objectives when complete virologic suppression is not possible, should be to maintain or restore immunologic function, stop the progression of the clinical illness, and minimize the emergence of new drug resistance that could further restrict the options for ARV drugs. Treatment history and drug-resistance testing, including the findings of previous and ongoing resistance tests, should be considered while selecting ARV regimens. Hence, the treatment approach post-ARV failure can be personalized based on clinical, immunologic, virologic, or as a mix of the three domains on a case-to-case basis. The evaluation of projected ARV activity should be based on treatment history and previous resistance test findings.

## Introduction and background

The global fight against the human immunodeficiency virus (HIV) has advanced remarkably during the past few decades. There is an increasing trend in a few countries in new infections when previously in decline. There has been an unprecedented increase in the use of antiretroviral (ARV) therapy (ART) in the last decade. It has prevented tens of millions of HIV-positive people from losing their lives. Of the projected 38.4 million people living with HIV globally, 28.7 million were taking ART by the end of 2021 [1]. Among the 30 surveys reported to the World Health Organization (WHO), pretreatment resistance to nevirapine (NVP) or efavirenz (EFV) in populations initiating first-line ART exceeded 10% in 21 instances. Additionally, pretreatment resistance to the non-nucleoside reverse transcriptase inhibitors (NNRTI) drug class is notably more prevalent, up to three times, in individuals with previous exposure to antiretroviral drugs. Concerningly, nearly half of infants born to mothers infected with HIV display resistance to one or more NNRTIs [1]. By 2025, 95% of all people living with HIV (PLHIV) should have received a diagnosis, 95% of them should be receiving life-saving antiretroviral therapy (ART), and 95% of PLHIV receiving treatment should have their viral load suppressed for both personal health reasons and to prevent HIV from spreading to others [2]. HIV has no cure and lifelong treatment is required. Thus, preventing ART failure in HIV-positive people could pose a major barrier to an effective treatment strategy.

Antiretroviral treatment failure is defined based on clinical, immunological, and virological measures. Viral suppression remains the key to the success of ART. The advantages of ART-induced immunological reconstitution are diminished by its failure, which also raises morbidity, and adverse outcomes related to HIV, and lowers the quality of life for HIV patients [3].

Patients with ART failure are becoming more common in countries with low resources, but estimates imply that only a small percentage of them are really diagnosed and embarked upon second-line ART; the majority are probably failing virologically but haven't been switched to a different regimen [4]. Meanwhile, higher coverage of ART in people living with HIV has led to a surge in the number of patients not responding to first-line treatment with continued ART usage [5]. However, due to the challenges involved in diagnosing the condition and the various reasons why ARV failure occurs, the burden of treatment failure is not extensively documented [6].

HIV drug resistance, inadequate dose, drug interactions, poor adherence, and inadequate medication absorption are important contributing factors that lead to antiretroviral (ARV) therapy failure that should be recognized and addressed [7]. To overcome this precarious predicament, a more effective approach must be adopted. Therefore, this review aims to highlight these factors for ARV failure and various approaches to combating this issue.

## Review

Factors contributing to ARV failure

**Figure 1:**
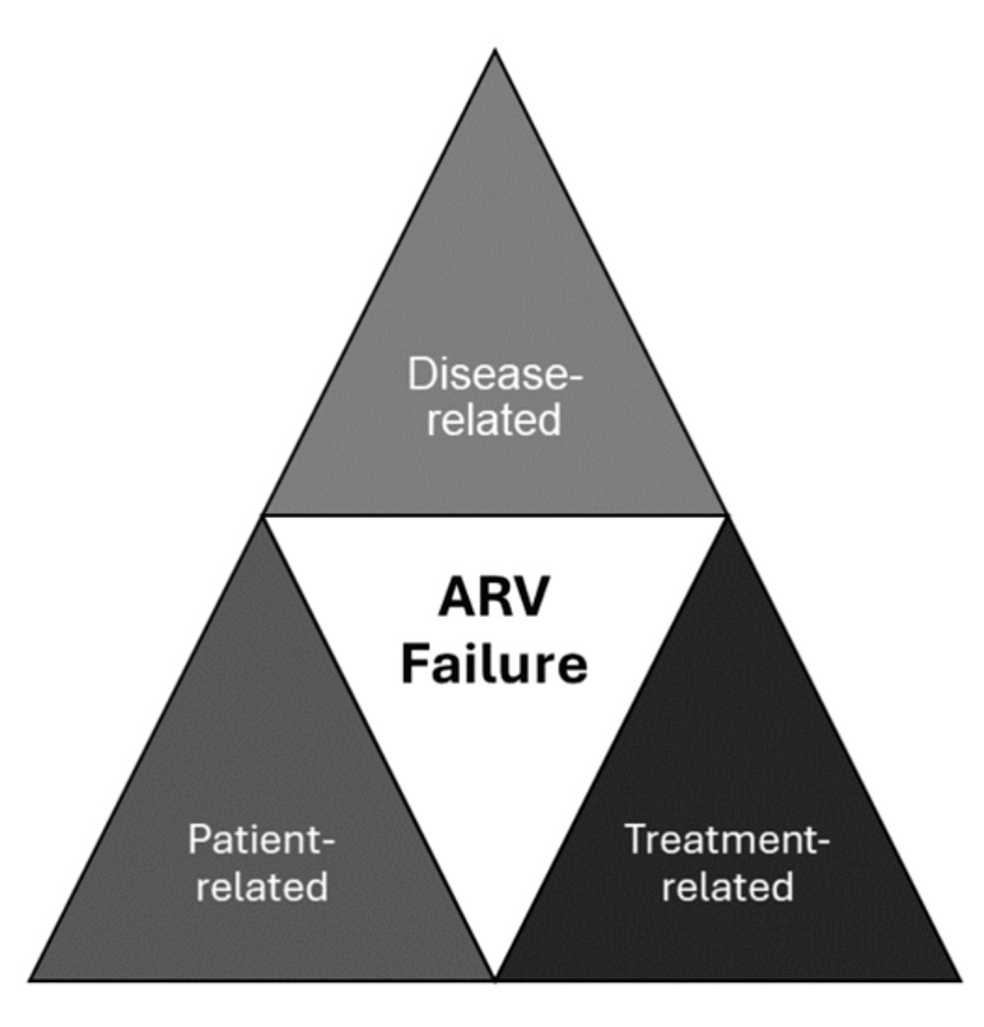
Factors contributing to ARV failure (Abbreviation: ARV = antiretroviral)

The contributing factors of ARV failure can be categorized as disease-, patient- or treatment-related factors (Figure 1). 

Disease-related factors

The possibility of acquired drug resistance or transmission of resistant virus poses a major risk for ARV failure [8]. It is difficult to diagnose but needs to be investigated in real-world clinical practice while treating HIV-positive individuals. There could be a probability of innate drug resistance to ART prescribed due to genetic variability. A moderate prevalence of transmitted drug resistance among ART-naïve patients with primary HIV infection was demonstrated [9]. Therefore, it has been suggested that baseline drug resistance testing is crucial for public health surveillance and for ART selection. Standard-prescribed ART could be ineffective against resistant HIV and higher pre-treatment viral RNA levels [10].

Patient-related factors

ART is a lifelong commitment and adherence to the treatment plays a pivotal role in the success of the therapy. The factors affecting adherence like comorbidities like substance abuse, psychological impairment, and mental disorder could affect the targeted outcomes [11]. Noncompliance to treatment follow-ups and interrupted or intermittent access to the ART could prove to be an obstacle to immune restoration and can lead to virologic failure [12].

Treatment-related factors

Antiretroviral drugs have the unique ability to combat a potentially highly mutagenic virus, i.e., HIV [13]. Any suboptimal dose regime may lead to therapeutic failure by affecting the desired therapeutic concentration and pharmacokinetic variability [14]. Drug-drug or drug-food interactions with or without prescription error may add to ARV failure [15]. Adverse drug reactions (ADRs) with antiretroviral drugs often hinder the treatment continuation [16]. Combined antiretroviral therapy has overcome problems related to pill burden and dosing frequency. Causality assessment of the offending drug for ADRs in combined therapy becomes a difficult task due to the inability to selectively dechallenge a particular therapeutic agent [17].

Approach to managing ARV failure

A thorough assessment of one or the other contributing factors for ARV failure is expected from a healthcare provider. The obvious causes for the treatment failure need to be elucidated to pave the way for the possible subsequent treatment approaches.

Approach to selecting ART regimen in virological failure

Treatment-related and other contributing factors should be contemplated to propose the most potent, efficacious, and well-tolerated regimen. A personalized treatment needs to be provided based on the patient’s history, drug resistance pattern, and ARV less susceptible to the resistance [18]. Fully susceptible antiretroviral drugs for patients of virologic failure could be drugs not previously selected against the resistant virus, new in class drugs, or antiretroviral drugs with novel mechanisms of action like capsid inhibitors, glycoprotein-120 attachment inhibitors, CCR5 antagonists, fusion inhibitors, or the latest post attachment inhibitor [19]. There could be a possibility of cross-resistance with the same class of drug previously prescribed resulting in partial or no activity against the resistance virus.

Approach to investigating drug resistance

ART for the ARV failure should be guided by drug resistance testing. As soon as it becomes evident that a patient is on a failing regimen and is still taking it, or at least four weeks after discontinuing, drug resistance testing should be carried out to obtain an understanding of resistance mutation. Drug resistance testing is best recommended for patients with virologic failure and HIV-RNA levels of more than 1000 copies /ml but even as low as 200 copies/ml should obtain test results [20]. Genotypic testing is preferred over phenotypic testing for known resistance patterns while phenotypic test results are also performed for suspected complex resistance mutation patterns [21].

Approach to designing new antiretroviral regimens

Two fully active medications, such as the boosted protease inhibitors (PI) darunavir (DRV/r) or the second-generation integrase strand transfer inhibitors (INSTI) dolutegravir (DTG), can be included in a new ARV regimen if at least one of them has a strong resistance barrier [22]. If both are completely active, a new ARV regimen can consist of an INSTI (preferably the second-generation DTG) and enhanced protease inhibitors (preferably boosted DRV), without NRTIs. The objective is to incorporate three totally active medications into the regimen if there is no agent with a high resistance barrier and is fully active. Some ARV medications in the regimen may still have some effect on the patient's HIV infection even in the presence of drug-resistance mutations and thus may be kept as part of a salvage strategy. These medications may include nucleoside/nucleotide reverse transcriptase inhibitors (NRTIs), PIs, and second-generation INSTIs [23]. However, when treating patients with relevant resistance mutations, the dosage of some medications (e.g., DRV and DTG) may need to be increased in order to achieve the drug concentrations required to be at least partially active against a less sensitive virus [24]. Conversely, it is best to stop using other medications when resistance is likely to develop as it is uncertain that using them longer will result in virologic suppression [25]. NNRTIs, particularly efavirenz, nevirapine, and rilpivirine (RPV), as well as raltegravir (RAL) and elvitegravir (EVG), the first-generation INSTIs, may be among these medications. Following a regimen changeover, patients should be regularly watched for virologic responses (e.g., HIV viral load testing within 4 to 8 weeks), and if virologic response is insufficient, drug-resistance testing should be performed without any delay [26] (Table 1).

**Table 1:** Antiretroviral regimen in Virologic Failure (Abbreviations: ART = antiretroviral therapy; ARV = antiretroviral; BIC = bictegravir; DRV = darunavir; DTG = dolutegravir; INSTI = integrase strand transfer inhibitor; NNRTI = non-nucleoside reverse transcriptase inhibitor; NRTI = nucleoside reverse transcriptase inhibitor; PI = protease inhibitor)

Clinical Scenario	ARV failed	New Regimen Options [27]
First Regimen Failure	NNRTI + two NRTIs	Two NRTIs + DTG (or possibly BIC)
	Boosted PI + two NRTIs	Two NRTIs +DTG (or possibly BIC)
	INSTI + NRTIs	•Two NRTIs+ Boosted PI •Two NRTIs+ DTG or BIC •Boosted PI + DTG
Second Regimen Failure and beyond (Use past and current genotypic- +/– phenotypic-resistance testing and ART history when designing new regimen)	Boosted PI, but not second-generation INSTI, fully active	Two NRTIs with boosted PI (ideally at least one completely active)
	Second-generation INSTI, but not boosted PI, fully active	Two NRTIs (ideally at least one completely active) combined with DTG or BIC
	Both PI and INSTI fully active	Boosted PI with INSTI or above two options
ART-Experienced Patients With Suspected Drug Resistance and Limited or Incomplete ARV and Resistance History	Unknown	Initiation of ARV regime with high genetic barrier to resistance if no ARV history is available (for example: DTG, BIC, and/or boosted DRV) with early resistance testing and close follow-up of virologic response

Individualized management strategy for virologic failure

A patient's ART exposure history, level of plasma viremia, duration of virologic failure, and degree of drug resistance can all be taken into consideration when designing an individualized therapy plan for ARV failure patients [28]. Ascertaining the amount of HIV viremia, assessing and dealing with adherence, and addressing any drug-drug (including interactions with over-the-counter medicines and supplements) and drug-food interactions are the initial steps for all patients with measurable viral loads. Hence, the decision on switching one ARV agent in a regimen to another agent in the same drug class or switching from one drug class to another class should be made on a case-to-case basis. If the current regimen is well tolerated with no food or drug interaction identified, it’s better to continue the same regimen with a focus on adherence [29]. If new or previously detected resistance mutations compromise the regimen, the regimen should be modified as soon as possible to avoid progressive accumulation of resistance mutations. It is best to prescribe the modified regimen before viremia worsens or before the CD4 count declines [30]. Patients with low viremia, defined as <200 copies/mL, should adhere to their current treatment plans and get their HIV-RNA levels checked at least every three months to determine if they may eventually have to modify ART [31]. Drug-resistance testing should be considered on patients who exhibit persistent plasma HIV-RNA levels between 200 and 1,000 copies/mL, as this is regarded as virologic failure. Suboptimal adherence is nearly invariably linked to HIV RNA ≥1,000 copies/mL and no drug-resistance mutations were found using genotypic resistance test findings from the past or present [32]. The patient's ability to get ART, the degree and duration of adverse medication responses, and the patient's tolerance to the present regimen can all be used to identify the variables contributing to inadequate adherence.

## Conclusions

Establishing virologic suppression is the main objective of therapy for individuals who previously received ART and have suffered virologic failure. Expert counsel is often needed to build virologically suppressive regimens for individuals who have suffered virologic failure after starting antiretroviral therapy (ART). It is crucial to thoroughly assess the cause(s) of virologic failure before making any changes to a regimen. These include inadequate adherence, poor tolerability, drug-drug and drug-food interactions, as well as changes in plasma HIV-1 RNA or HIV-1 viral load and CD4 count over time, a thorough treatment history, and the results of both recent and past drug-resistance tests. In the rare circumstance that virologic suppression remains unattainable, the regimen being considered needs to prioritize toxicity mitigation as well as upholding therapy alternatives, all the while keeping CD4 counts to impede clinical progression.
